# Mature Cystic Renal Teratoma

**DOI:** 10.5812/iranjradiol.11260

**Published:** 2014-01-30

**Authors:** Alpaslan Yavuz, Kagan Ceken, Emel Alimoglu, Bahar Akkaya

**Affiliations:** 1 Department of Radiology, Yuzuncu Yil University Hospital, Ercis Yolu, Geve Kampus, Van, Turkey; 2 Department of Radiology, Akdeniz University, Kampus Antalya, Turkey; 3 Department of Pathology, Akdeniz University, Kampus Antalya, Turkey

**Keywords:** Teratoma, Ultrasound, Computed Tomography, Cystic, Kidney

## Abstract

Teratomas are rare germline tumors that originate from one or more embryonic germ cell layers. Teratoma of the kidney is extremely rare, and less than 30 cases of primary intrarenal teratomas have been published to date. We report the main radiologic features of an unusual case of mature cystic teratoma arising from the left kidney in a two-year-old boy. A left-sided abdominal mass was detected on physical examination and B-Mod Ultrasound (US) examination revealed a heterogeneous mass with central cystic component. Computed tomography (CT) demonstrated a lobulated, heterogeneous, hypodense mass extending craniocaudally from the splenic hilum to the level of the left iliac fossa. Nephrectomy was performed and a large, fatty mass arising from the left kidney was excised. The final pathologic diagnosis was confirmed as cystic renal teratoma.

## 1. Introduction

Teratomas are neoplasms that originate from one or more embryonic germ cell layers. The primary involvement site is the gonadal tissue; less frequent primary sites of involvement include the anterior mediastinum, retroperitoneum, sacrococcygeal region, brain and the gastrointestinal tract. Teratomas of the kidney are extremely rare, and less than 30 cases of primary intrarenal teratomas have been published to date. In some cases, renal teratomas have been mistaken as teratoid nephroblastomas (1). In most of the cases considered as renal teratoma, the final diagnosis was either retroperitoneal teratomas with renal extension or Wilms’ tumor with teratoid components (2). We present the main radiologic features of a renal teratoma case in the context of the current literature.

## 2. Case Presentation

**Figure 1.**
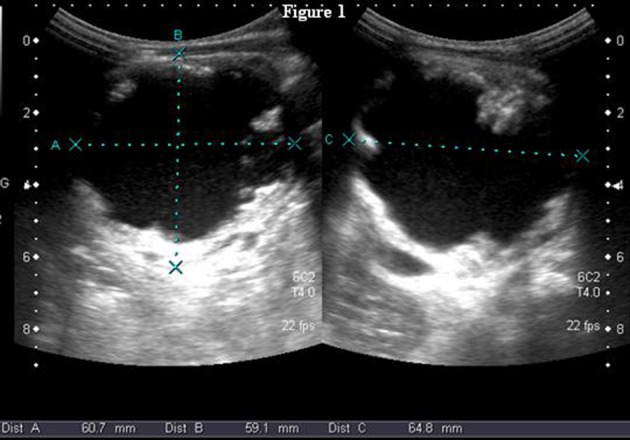
B-Mode US scan of the heterogeneous, cystic-solid mass involving the entire left kidney. The size of the lesion in three planes is 60×59×64 mm.

**Figure 2.**
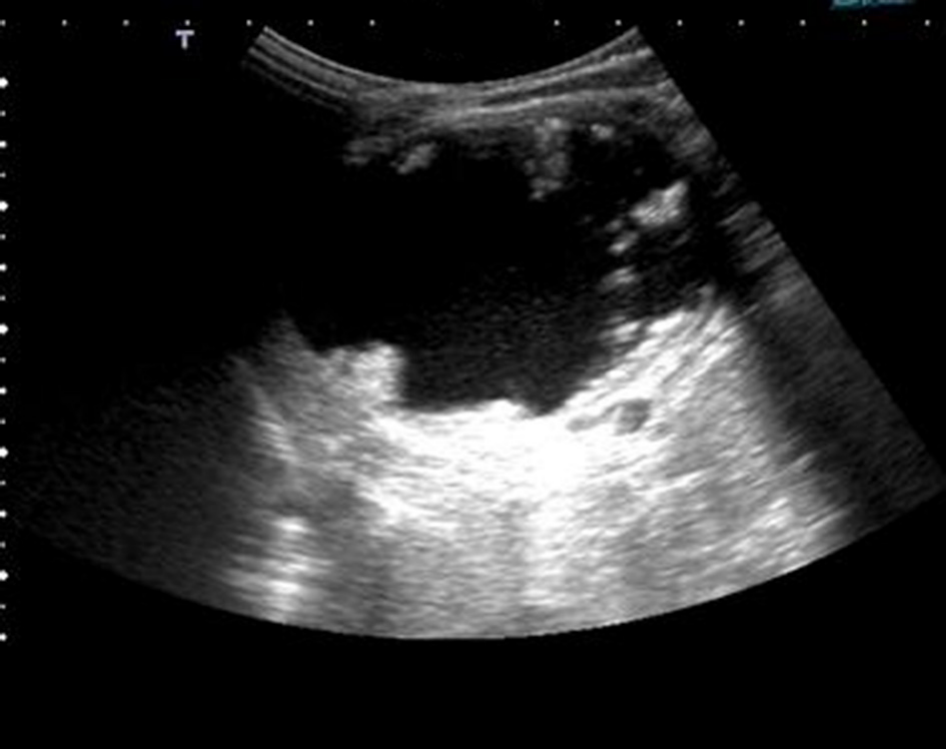
Sonographic posterior acoustic enhancement of the semi-solid cystic mass is clearly seen.

**Figure 3.**
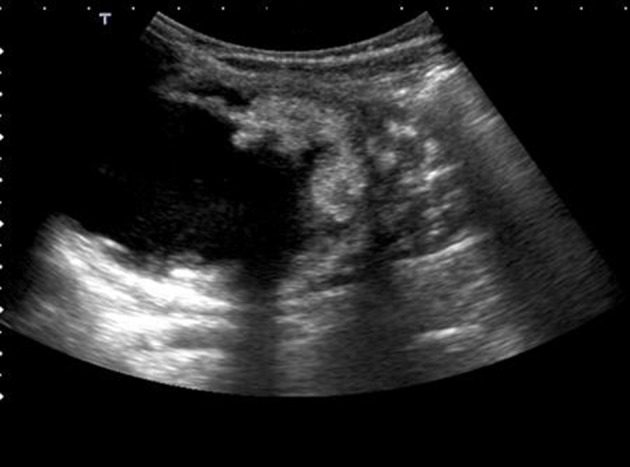
Nodular protrusion of the solid component towards the central region and anechoic-cystic central component is seen. Calcifications are not remarkable in US.

**Figure 4.**
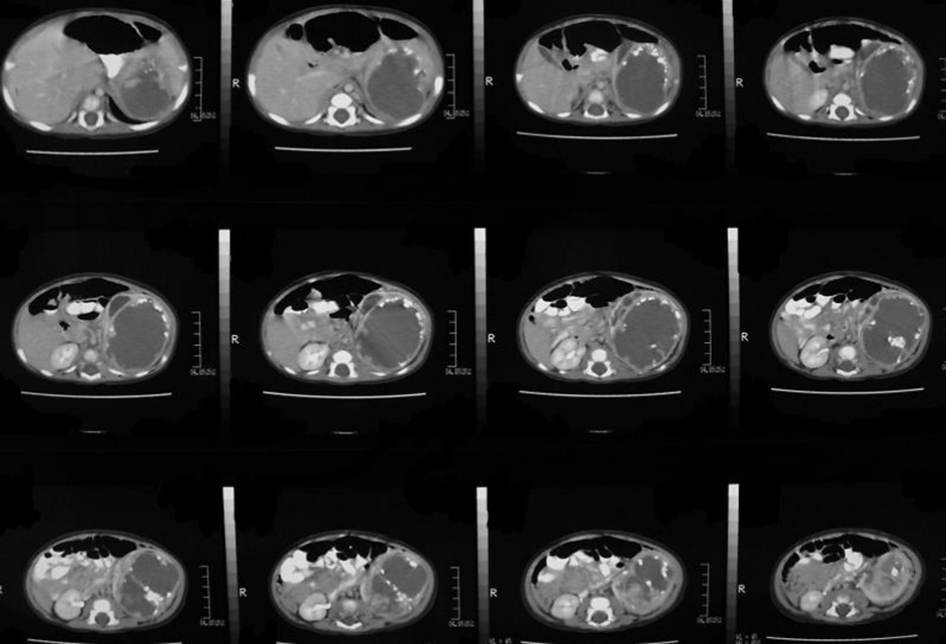
Helical CT examination of heterogeneous, cystic renal mass within the left retroperitoneal site shows peripheral fatty tissue deposits with low attenuation values.

**Figure 5.**
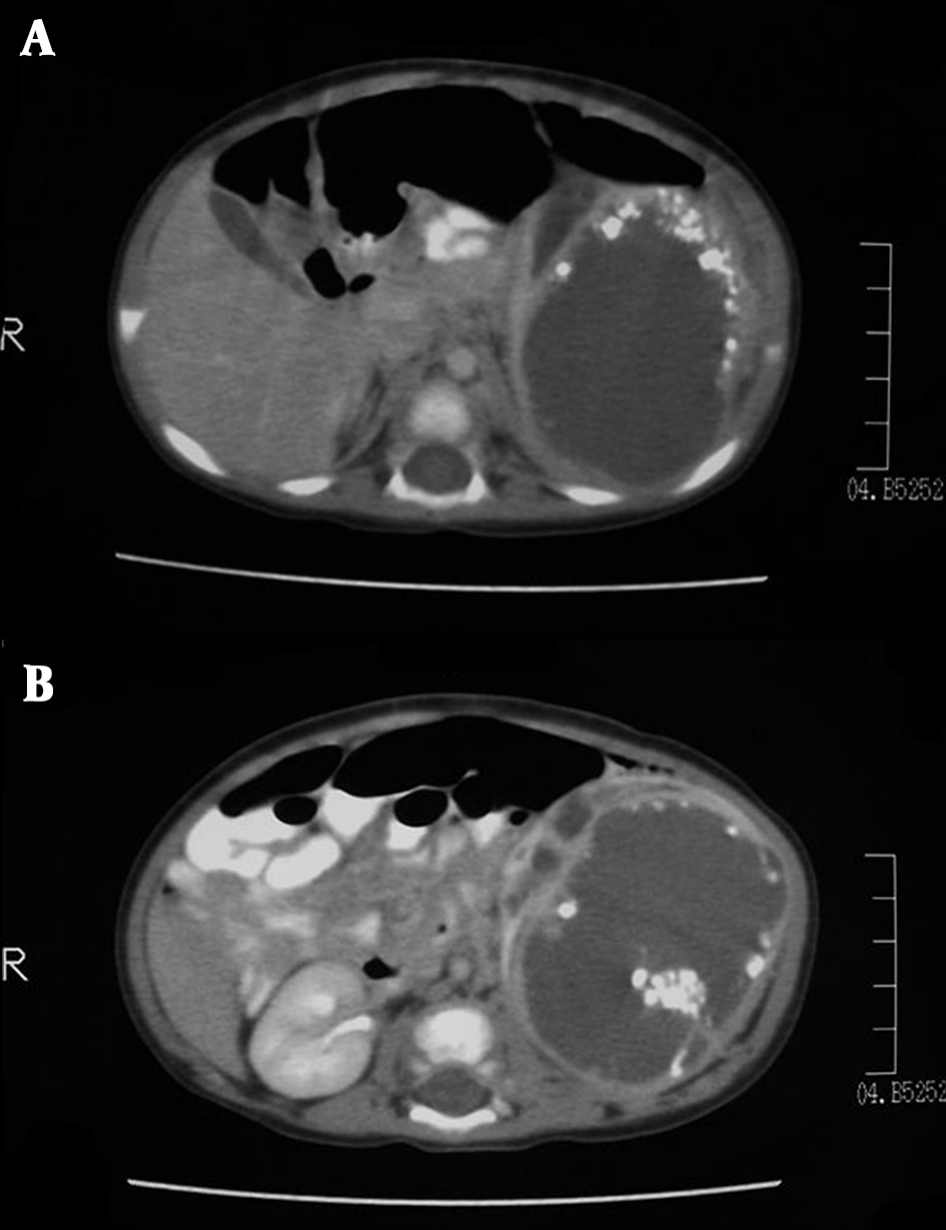
A, Peripheral coarse calcifications on CT. B, Calcifications are clustered in particular regions of the mass.

A two-year-old boy with a left-sided abdominal mass on physical examination was admitted to our clinic. Ultrasonography revealed an ovoid shaped 60×59×64 mm ^3^ sized, heterogeneous mass consisting of intermediate echogenicity (Figure 1). The central region was composed of an irregular hypoechoic component with posterior acoustic enhancement (Figure 2). Faint nodular protrusions from the peripheral solid component into the hypoechoic center were seen (Figure 3). The mass had no significant vascularity in color or power Doppler US. CT demonstrated a lobulated, heterogeneous, hypodense mass extending craniocaudally from the splenic hilum to the level of the left iliac fossa (Figure 4). Peripheral hyperdense coarse calcifications and adipose tissue components were well demonstrated by CT, with clusters of calcific foci (Figure 5). Nephrectomy was performed and a large, fatty mass arising from the left kidney was excised. The final pathologic diagnosis was confirmed as cystic renal teratoma.

## 3. Discussion

The most common renal neoplasms in the pediatric age group are nephroblastoma, mesoblastic nephroma, clear-cell sarcoma and rhabdoid tumors. Other primary neoplasms include primitive neuroectodermal tumor (PNET), renal cell carcinoma and angiomyolipoma. In childhood, diagnostic problems frequently occur especially when tumors contain a variety of heterogeneous elements. This is the main reason for misdiagnosis of teratomas. Differentiation of teratoma and other renal neoplasms is often problematic (3). The preoperative differential diagnosis of children with an intrarenal teratoma includes Wilms’ tumor, neuroblastoma, hydronephrosis, retroperitoneal teratoma, unspecified tumor, and infected renal cysts. For adults, the list includes unspecified tumors and renal cell carcinoma (4). Teratomas are usually benign. Malignant metastases have been reported in some cases with well-differentiated teratomas of various organs, thus teratomas arising in the kidney have the potential of metastatic spread (5). There is no report presenting distant metastasis from a mature teratoma of the kidney, but this could be because of its rarity and we also did not determine any metastatic activity in our case.

Choi et al. (4) reported a literature review summary of primary renal teratomas from 1934 to 2005. Twenty cases were included in this report. The statistical distribution of these cases was reported as twelve renal teratomas (60%) found in children and eight (40%) in adults (average age, 17 years; median age, 3 years). The female-to-male ratio was about 1.4:1 (1.8:1 in children; gender was not reported in one pediatric case). Fifteen teratomas (75%) were of immature histologic grade, and of these tumors, just less than 50% were locally infiltrating or metastatic (similar to children). Associated congenital abnormalities (horseshoe kidney, duplicated collecting system, prune belly syndrome and oligodactyly-like syndrome) were seen in five cases suggesting that maldevelopment increases the risk of teratoma. In fact, horseshoe kidney has been associated with an increased risk of Wilms’ tumor, renal pelvis cancer (2, 6), and primary renal carcinoid tumor (7). There are a few other case reports in the literature presenting teratomas arising from the kidney. Glazier et al. (8) reported a left sided renal cystic teratoma on ultrasonography in a 59-year-old woman. Ishii et al. published the CT findings of a case with a large teratoma originated from the right kidney in a 3-month-old girl and differential diagnostic properties of the entity from Wilms’ tumor were discussed (9). Mohindra et al. (10) reported a unique case of renal intramedullary mature cystic teratoma presenting as isolated renal failure. Another interesting relationship between primary carcinoid tumors and renal teratomas has been revealed by several case reports. Five cases of renal teratoma associated with carcinoid tumor have been reported to date (2, 6, 11, 12). Primary carcinoid tumors arising within these five renal teratomas all occurred in adult patients. In our case, no symptoms related to carcinoid syndrome were seen and no carcinoid component was apparent in the pathologic evaluation. On radiologic examinations, intrarenal teratomas often manifest as large, unilateral abdominal masses, sometimes with calcifications, as in the case presented. Ultrasound patterns include cystic, heterogeneous, mixed cystic-solid and hyperechoic patterns with occasional coarse foci of calcifications (4). In our case, the mass could be classified in the cystic-solid group by ultrasound; however, calcifications were sonographically unremarkable. CT examination clearly demonstrated the various tissue components of the tumor such as fat, calcifications and cysts. In the literature, the radiologic appearance of renal teratoma has been reported as heterogeneous masses, sometimes with cystic areas, coarse foci of calcifications or necrosis. These imaging features have been reported for extragonadal teratomas in other organs (4). One case of renal teratoma was reported with MRI imaging characteristics and described the mass as heterogeneous with primarily low signal intensity in T1-weighted sequences and high signal intensity in T2-weighted sequences (13). Primary teratoma of the kidney is exceptionally rare. However, teratomas must be included in the differential diagnosis of large, cystic, heterogeneous renal masses especially if the coexistence of peripheral coarse calcifications, fatty tissue components or bone-like structures is seen.
